# All-optical extravascular laser-ultrasound and photoacoustic imaging of calcified atherosclerotic plaque in excised carotid artery

**DOI:** 10.1016/j.pacs.2018.01.002

**Published:** 2018-02-07

**Authors:** Jami L. Johnson, Mervyn Merrilees, Jeffrey Shragge, Kasper van Wijk

**Affiliations:** a University of Auckland, Faculty of Science, Department of Physics, Dodd-Walls Centre for Photonic and Quantum Technologies, Private Bag 92019, Auckland 1010, New Zealand; b University of Auckland, Faculty of Medical and Health Sciences, Department of Anatomy and Medical Imaging, Private Bag 92019, Auckland 1142, New Zealand; c Colorado School of Mines, Center for Wave Phenomena, Geophysics Department, Golden, CO, USA

**Keywords:** Atherosclerosis, Photoacoustic imaging, Laser-ultrasound, Calcification, Reverse-time migration

## Abstract

Photoacoustic (PA) imaging may be advantageous as a safe, non-invasive imaging modality to image the carotid artery. However, calcification that accompanies atherosclerotic plaque is difficult to detect with PA due to the non-distinct optical absorption spectrum of hydroxyapatite. We propose reflection-mode all-optical laser-ultrasound (LUS) imaging to obtain high-resolution, non-contact, non-ionizing images of the carotid artery wall and calcification. All-optical LUS allows for flexible acquisition geometry and user-dependent data acquisition for high repeatability. We apply all-optical techniques to image an excised human carotid artery. Internal layers of the artery wall, enlargement of the vessel, and calcification are observed with higher resolution and reduced artifacts with nonconfocal LUS compared to confocal LUS. Validation with histology and X-ray computed tomography (CT) demonstrates the potential for LUS as a method for non-invasive imaging in the carotid artery.

## 1 Introduction

Stroke is currently the second leading cause of death and morbidity worldwide [1]. These cerebrovascular events result from atherosclerotic plaque deposits rupturing and forming blood clots that occlude blood flow to the brain. Therefore, both understanding and preventing carotid atherosclerotic disease is of substantial interest [2]. Certain characteristics of plaque deposits can contribute to rupture vulnerability [[3], [4]]. Accepted factors include a thin, fibrous cap (<100 μm [3]), spotty calcification [[5], [6]], positive remodeling, a large lipid core (>40% plaque volume) [3], and intraplaque neovascularizations [4]. Biomedical imaging of the carotid artery is therefore of primary importance for determining disease risk, preparing for surgical intervention, and monitoring treatment outcomes. Favorable characteristics of carotid imaging include accurate, high resolution, repeatable, and operator-independent capabilities that facilitate diagnosis and treatment in a rapid time window with minimal risk [2]. Furthermore, imaging that is practical for screening and allows for longitudinal studies to better understand cardiovascular disease is desirable [6].

Calcification, in particular, not only contributes to plaque vulnerability, but is also a concern for many additional cardiovascular diseases and conditions. Examples include calcification of vascular implants (valves [7], grafts [8], and stents [9]), post surgery calcification [10], and vascular calcification in hemodialysis patients [[11], [12]].

A range of imaging modalities are currently used to assess vulnerable characteristics of atherosclerotic plaque in the carotid artery (Table 1). Each modality has advantages and limitations depending on the clinical requirement. In general, intravascular modalities offer superior resolution compared to non-invasive imaging due to proximity to the target and/or contrast enhancement. Nonetheless, non-invasive modalities are often the first line of assessment, and in some cases a combination of non-invasive modalities are used exclusively for diagnosis [2]. Ultrasound (US), computed tomography (CT), and magnetic resonance imaging (MRI) are capable of imaging several characteristics of atherosclerotic plaque non-invasively, including calcification. Magnetic resonance imaging (MRI) has the ability to image a range of components with sub-millimeter resolution, but the high cost, low signal-to-noise, and motion artifact will likely limit MRI for widespread plaque screening [13]. In CT, calcifications may be masked by radiopaque contrast in the vessel lumen [14], and CT cannot differentiate between intimal and medial calcification [[15], [11]], the former of which has been shown to destabilize plaque [16]. “Blooming” artifacts are also common to CT images of calcification and cause significant overestimation of calcified plaque (average of 400% for multi detector CT of the coronary artery) [17]. Additionally, CT uses ionizing X-ray radiation that is undesirable for screening. A recent study of calcification in thyroid nodules found that ultrasound is more sensitive to calcification than CT [18]. Nonetheless, CT is currently the gold-standard for calcification detection [[11], [12]]. Calcification has strong acoustic contrast compared to soft tissue, and the relative low-cost and safety of US are desirable for plaque screening. However, operator skill is known to cause inter-operator variability in US imaging [19] whereas CT and MRI use fixed, remote acquisition geometries that are well-suited to follow-up studies.

**Table 1:** Imaging modalities used to detect various characteristics of vulnerable atherosclerotic plaque in the carotid artery: magnetic resonance imaging (MRI), ultrasound (US), intravascular US (IVUS), contrast enhanced US (CEUS), optical coherence tomography (OCT), computed tomography angiography (CTA), multi detector CT (MDCT), and positron emission tomography (PET). This table is modified from Ibrahimi et al. [6].

Plaque feature	Imaging modality
Thin cap	IVUS, OCT, MRI
Positive remodeling	MRI, CTA, IVUS
Large lipid core	US, MDCT
Plaque composition	US, MDCT
Neovascularization	CEUS
Intraplaque hemorrhage	MRI
Inflammation	PET
Calcification	US, CT, MRI, IVUS

Herein, we present laser-ultrasound (LUS) imaging as a candidate for non-invasive imaging of the carotid artery and associated calcification. Like US, LUS provides improved details of the artery wall and the location of calcification within the artery wall compared to CT, while achieving operator-independent, highly repeatable data acquisition capabilities. As LUS uses non-ionizing radiation, it may also be suitable for screening and longitudinal studies. Further, the achievable pulse-width and lateral resolution are improved with all-optical systems compared to piezoelectric transducers, and the quantitative nature of optical detectors open up the potential to create quantitative maps of acoustic properties in the tissue with non-ionizing radiation.

### 1.1 Photoacoustic and laser-ultrasound imaging

**Fig. 1:**
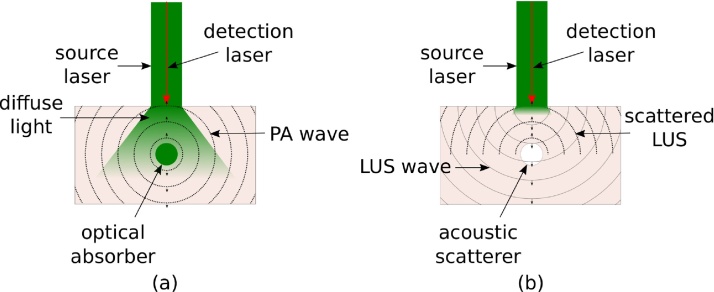
Diagram of (a) photoacoustic (PA), and (b) laser-ultrasound (LUS) generation, wave propagation, and optical detection. In (a) light propagates deep in tissue. A PA wave is generated upon absorption by an optical absorber. The PA wave propagates to the surface, and the resulting surface displacement is recorded by an optical detector. Strong absorption of light occurs at the surface of tissue to generate an LUS wave in (b). The LUS wave is scattered/reflected back to the surface by acoustic inhomogeneities, where it is detected. Arrows indicate the direction of propagation of the wavefronts.

PA imaging maps optical absorption properties of tissue up to centimeters deep, overcoming the diffusion-limited imaging depths of purely optical imaging modalities, such as OCT. A nanosecond-pulse of light rapidly becomes diffuse upon propagating through highly scattering biological tissue. Chromophores in the path of the diffuse beam absorb the light, causing thermoelastic expansion and the generation of pressure waves originating at the location where the majority of light is absorbed (Fig. 1(a)). Detection and localization of these acoustic sources create a PA map of optical absorption.

PA imaging has proven sensitive to the optical absorption contrast of both lipids and hemoglobin in the carotid artery. PA systems have demonstrated imaging depths of 2 cm in tissue phantoms [20] and 3.5 cm *in vivo* with intrinsic contrast [21]. Further, Dima et al. [22] showed that PA imaging of the carotid artery is possible *in vivo* at depths of 2 cm using both linear and curved transducer arrays. Deep-tissue imaging of the carotid artery primarily utilizes the intrinsic contrast of hemoglobin to image the vessel structure. Hemoglobin is a strong optical absorber in the optical window (∼600 to 900 nm), where light is weakly absorbed by skin. Near-infrared light is preferred for PA imaging of lipids [23], however, near-infrared is strongly absorbed by skin and subcutaneous fat. Therefore, non-invasive light delivery for lipid plaque detection may not be possible. Light delivery through the pharynx is a promising approach for non-invasive imaging of lipid pools in the carotid artery wall closest to the pharynx [24], but intravascular light delivery may be required for illumination of the wall closest to the skin surface.

Detecting calcification is not straightforward with PA, as the optical spectrum is not unique in the visible and near-infrared wavelength range [25]. It is known that US is sensitive to calcification, yet enhanced resolution and reduced inter-operator variability are desirable for reliably detecting calcification deposits. In contrast to transducer-based US, LUS uses the photoacoustic effect at the tissue surface (Fig. 1(b)) to create broadband, highly repeatable acoustic sources without the need for contact with the sample or a coupling agent.

LUS images of acoustic reflectivity [[26], [27]] and speed-of-sound [[28], [29]] have been demonstrated, both of which are complemented by combining with PA imaging. Furthermore, the information obtained by LUS can be used to map acoustic density and velocity inhomogeneities [30] or measure the attenuation field that can then be used in reconstruction of PA images to reduce artifacts. The synergy of PA and LUS imaging is further evidenced by the ability to use the same acquisition system for both techniques. Water is weakly absorbed in the optical window, thus PA generation dominates in this range. LUS dominates when wavelengths beyond 1000 nm are used, because most optical energy is absorbed at the surface of tissue by water. Therefore, we can alternate between PA and LUS generation by changing the source laser wavelength or surface optical properties of the sample [26]. The properties of the LUS wave can be further enhanced by designing an absorbing layer with optical and elastic properties that produce enhanced power or bandwidth [[31], [32]].

We propose an all-optical system that utilizes optical detection of the LUS and PA wavefields. Conventional US requires a contacting probe to generate acoustic waves in a narrow frequency band. The resonant behavior of piezoelectric elements elongates the pulse-width, limiting axial resolution [19]. In contrast, LUS wavefields are free of mechanical ringing (see Appendix A), and generally optical detectors have a broader frequency bandwidth and smaller spot size than transducer elements. The lateral resolution with all-optical systems is on-par with the highest resolution ultrasonic scanners, and can be even higher with the minimum, diffraction-limited spot sizes. The frequency content of laser-generated waves are broadband, and can reach tens of megahertz, therefore removing the need to change between high- and low-frequency probes. High-frequency components are attenuated quickly with depth, but are able to resolve superficial structures with high resolution. Lower-frequency components of laser-generated waves continue to propagate deeper, and can resolve larger structures. As both high- and low-frequency components are present in broadband LUS fields, the resolution of LUS images is ultimately limited at each depth by frequency-dependent attenuation.

The bandwidth, pulse-width, and spot-size of optical detectors enhance the lateral and axial resolution compared to piezoelectric techniques, however, sensitivity is typically reduced. For this particular application, the acoustic contrast of calcification is significantly higher than soft tissues, therefore, resolution is more important than sensitivity. Moreover, research and development of such optical detectors is active, and utilize the detection power of interferometery [[33], [27], [34]], Fabry-Pérot cavities [35], micro-ring resonators [36], optical beam deflection [37], and Fiber Bragg gratings [38]. The detection sensitivity is continually improving, and noise-equivalent displacements of about 0.2 kPa have been achieved [35]. Herein, we utilize laser-Doppler vibrometery [39] to measure the particle displacement at a focused spot at the sample surface, which offers a displacement sensitivity of 0.05 pm Hz^−1/2^ when detecting on a reflective tape (OFV-505, Polytec, Irvine, CA, USA). Furthermore, all-optical systems provide remote acquisition geometries for user-independent acquisition. It is also important to note that quantitative amplitude measurements can be obtained with optical detectors, and therefore quantitative maps of acoustic density are made possible with all-optical systems when coupled with an appropriate reconstruction algorithm.

In the following, we demonstrate all-optical nonconfocal LUS imaging of a fixed human carotid artery, and complementary photoacoustic imaging capabilities. Like CT and MRI, all-optical PA and LUS use non-contact, known acquisition geometries. Data are acquired independent of user factors, such as applied pressure and acoustic coupling, which are known to cause variations in transducer-based US. We use nonconfocal LUS imaging, where we independently control the source and detection beams to obtain angle-dependent images [40], and compare this to the standard confocal LUS approach. PA imaging of the carotid artery is increasingly studied, therefore, we focus on LUS imaging for calcification detection and imaging of the artery wall. However, we demonstrate the capabilities for dual-modality PA and nonconfocal LUS imaging by filling the artery with a phantom hemoglobin to generate PA waves. Finally, we validate the LUS images with histology and compare to CT.

## 2 Methods

### 2.1 Experimental setup

**Fig. 2:**
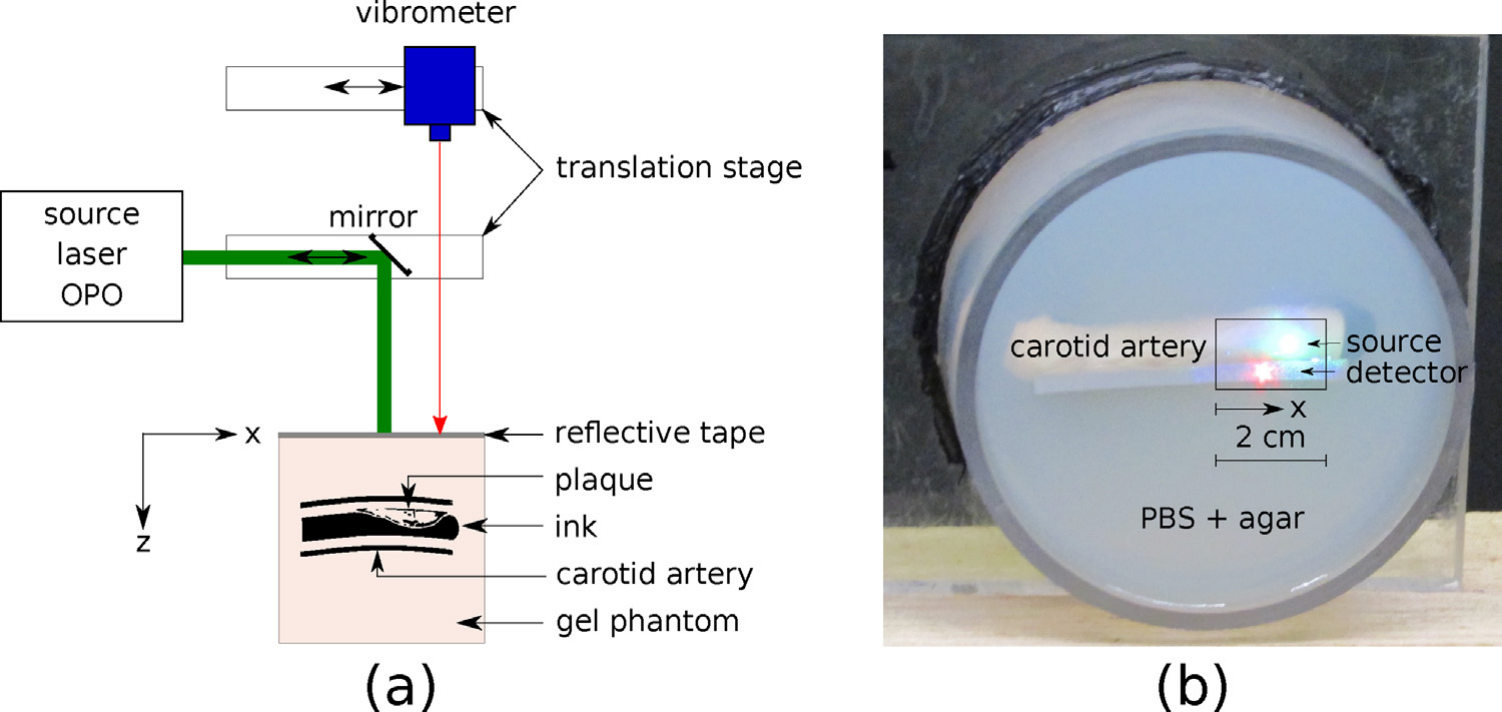
(a) Experimental setup for PA and LUS imaging of carotid artery. (b) Photograph of carotid artery sample embedded in phantom gel. The detection beam is incident on the reflective tape, while the source beam is incident on the phantom surface. The box indicates the location along the artery that is scanned.

The experimental setup is shown in Fig. 2. A human carotid artery (male, age 53) was collected at autopsy, pressure perfused at 120 mmHg and fixed in 4% paraformaldehyde and 2.5% glutaraldehyde in 0.1 M sodium cacodylate buffer at pH 7.4. The artery is embedded approximately 1 cm below the surface of a 1% agar and phosphate buffer solution phantom. The unique spectroscopic properties of biological tissues degrade when fixed in formalin, such that the absorption coefficient is close to zero [41]. Therefore, photoacoustic imaging of plaque components with unique spectroscopic properties (such as lipids or collagen) is not possible. Instead, we fill the artery with absorbing ink (1.6% India ink) with an absorption coefficient *μ*_*a*_ ≈ 70 cm^−1^ at 680 nm to mimic hemoglobin in the artery [42]. The absorption coefficient is comparable to oxygenated hemoglobin at this wavelength [43].

The all-optical PA and LUS system consists of a source and detection laser. The source laser beam (Radiant 532 LD, Opotek, Carlsbad, CA, USA) has a 5 ns pulse-width. The beam is collimated (5 mm diameter) and incident on a mirror mounted on a linear stage, which reflects the beam onto the phantom surface. A laser-Doppler vibrometer (OFV-505, Polytec, Irvine, CA, USA) is used to detect the resulting ultrasonic waves. The detector is mounted on a second linear stage. The source beam is directed perpendicular to the phantom surface and is incident on a retroreflective tape to enhance detection sensitivity. The source and detection beam are offset by 5 mm to allow the source beam to be incident directly on the phantom surface. Therefore, the beams are not coincident in the plane perpendicular to the beams, as shown in Fig. 2(b). We correct for this offset numerically (Section 2.2), however, we note that optical detectors exist that allow the source and detection beam to be coincident experimentally while maintaining adequate sensitivity [[35], [27]].

We independently control the motion of the source and detector beams by controlling the linear stages with PLACE software [44]. First, the source wavelength is tuned to 680 nm with a pulse energy of 20 mJ/cm^2^ for strong absorption by the ink and generation of PA waves as in Fig. 1(a). A confocal PA wavefield is recorded by scanning the source and detector beams simultaneously across 2 cm of the phantom surface at 200 μm increments. Second, a confocal LUS wavefield is recorded with a 1450 nm source beam and 40 mJ/cm^2^ pulse energy. LUS waves are generated preferentially at the tissue surface as in Fig. 1(b), analogous to the superficial generation of ultrasound waves by piezoelectric elements in B-mode imaging. The source LUS wavelength was chosen to be 1450 nm due to the high absorption coefficient of water at this wavelength [45], and favorable trade-off between amplitude and frequency content at depth. Appendix A details an optimization experiment to choose the LUS source wavelength. Finally, we record a nonconfocal LUS dataset by iteratively scanning the source and detection beam. The source beam is kept at a stationary location, while the detection beam is scanned across a 2 cm detection line at 200 μm increments. Then, the source beam is moved by 200 μm and the detection scan is repeated. This iterative process is continued for all 100 source positions. In this way, we obtain information about angle-dependent scattering and reflection of waves traveling between each source-detector pair [40]. For every detector position, the average of 32 waveforms is recorded with a 50 megasamples per second sampling rate.

To compare all-optical LUS imaging to state-of-the-art clinical imaging, a CT scan is acquired. A Phillips 128-slice scanner was used with an 80 kV energy and an average dose of 309 mA s. The minimum slice thickness of 0.2 mm for the sagittal and coronal plane, and 1 mm for the axial plane are used. Finally, histological imaging was performed for longitudinal slices of the artery stained with haemotoxylin and eosin.

### 2.2 Data processing and image reconstruction

Low-frequency air waves are filtered from the LUS and PA data with a 300 kHz highpass filter, and surface waves are muted. To correct for the offset between the source and detection beam, a normal moveout (NMO) correction is applied [[46], [47], [48]]. The confocal LUS image and PA image are reconstructed with time reversal, where the velocity is divided by two to account for two-way propagation time in the confocal LUS reconstruction. The nonconfocal LUS image is reconstructed with reverse-time migration [[49], [50]]. Details of the reconstruction techniques are described in [40].

## 3 Results

### 3.1 Laser-ultrasound images

**Fig. 3:**
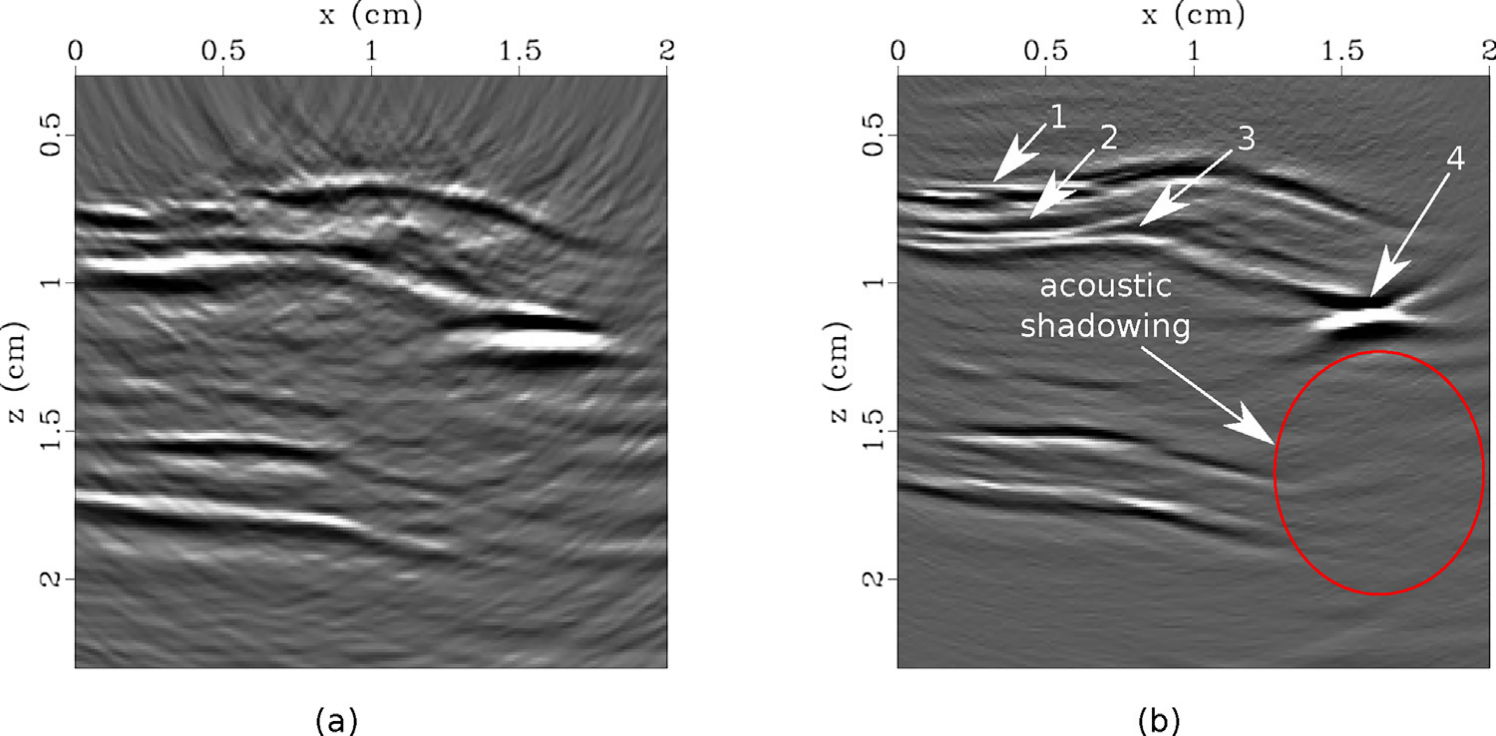
Laser-ultrasound images created with confocal (a) and nonconfocal (b) acquisition. Images are saturated to highlight the details of the artery wall. In (b), arrows 1–3 indicate the adventitia, media, and intima layers of the wall, and arrow 4 denotes calcification. Acoustic shadowing accompanies the calcification.

**Fig. 4:**
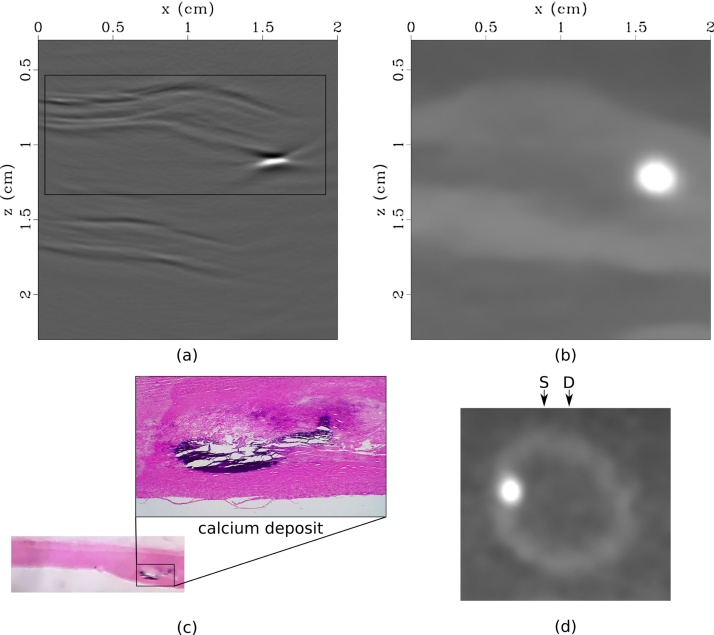
(a) Nonconfocal LUS image, (b) sagittal CT slice, (c) histological section, and (d) axial CT slice with LUS source (S) and detection (D) laser locations indicated. The box in (a) corresponds to the wall imaged for histology in (c). All images clearly indicate calcification in the upper wall, however (d) identified that the scatterer is outside of the acquisition plane.

Confocal and nonconfocal LUS images are shown in Fig. 3. In all images, a strong scatterer is seen at approximately (*x* = 1.6 cm, *z* = 1.2 cm) accompanied by an acoustic shadowing zone, indicating calcification. Furthermore, positive remodeling is evidenced by the increased thickness of the artery wall from approximately *x* = 0.75 cm to *x* = 1.5 cm. Both the confocal and nonconfocal LUS images reveal reflections by the adventitia and intima interfaces of the artery wall. However, the nonconfocal image shows additional details of the artery wall, including the media interface (Fig. 3(b)). Furthermore, the limited-view artifacts are suppressed, signal-to-noise is improved, and the strong scatterer is more focused in the nonconfocal image. The nonconfocal LUS image is shown with the corresponding CT slice and histology section in Fig. 4. All images clearly indicate the presence of calcification.

### 3.2 Photoacoustic imaging

**Fig. 5:**
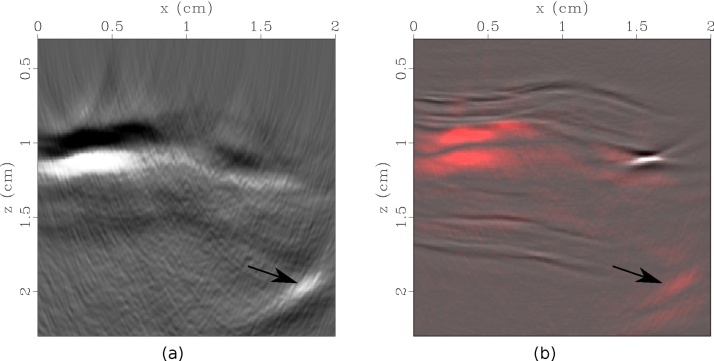
(a) Photoacoustic image of optical absorber representing haemoglobin (ink) in human carotid artery. (b) Absolute value of photoacoustic image (red) overlayed onto nonconfocal LUS image (grayscale). The arrows represent a reflection artifact caused by scattering of the PA wave by calcification.

The reconstructed PA image is shown in Fig. 5(a). Due to the large inner diameter of the artery, most of the source light is absorbed at the superficial interface between the intima of the artery wall and the ink, generating a low-frequency PA wave. Analysis of the PA image, alone, does not provide clear evidence of plaque in the artery. However, the PA signal is weaker from approximately *x* = 0.75 cm to *x* = 1.5 cm, indicating that the light attenuates through a thicker portion of tissue in this region.

## 4 Discussion

All-optical LUS imaging demonstrates potential for broadband, high-resolution ultrasonic imaging of the carotid artery wall and calcification deposits non-invasively. LUS is sensitive to the structure of the artery wall and acoustic scatterers, such as calcification, whereas PA imaging can be used to image optical absorbers, such as hemoglobin. Traditional US is sensitive to the same characteristics as LUS, however, the repeatability, resolution and flexibility limitations of US for this application motivate exploration of all-optical alternatives.

CT is the gold-standard for imaging calcification in the carotid artery, however, we observe that LUS is more sensitive to the layers of the artery wall. In Fig. 4, we also see improved lateral resolution in the nonconfocal LUS image compared to the CT image, which suffers from blooming artifact. However, due to the high acoustic contrast between hydroxyapatite and soft tissue, most of the LUS energy is reflected by the superficial surface of the calcification. The resulting acoustic shadowing does not allow imaging of structures below the calcification. This is characteristic of B-mode US as well. We note that the acoustic shadowing zone is reduced in the nonconfocal image compared to the confocal image. In this example, the calcification was located near the edge of the artery length. In a realistic situation where the vessel extends beyond the deposit, the aperture can be extended such that nonconfocal (angle-dependent) images can image below the deposit. This is analogous to the problem of imaging below a salt deposit in the earth in seismology, which has been successfully addressed by similar angle-dependent imaging techniques [51]. The acoustic shadowing zone may be reduced or even eliminated in this case. This highlights the flexibility of all-optical systems to dynamically tune the acquisition geometry.

Analysis of the PA image in Fig. 5(a), alone, does not provide clear evidence of plaque in the artery. However, the PA signal is weaker from approximately *x* = 0.75 cm to *x* = 1.5 cm, indicating that the light attenuates through a thicker portion of tissue in this region. The combination of PA and LUS imaging provides a more comprehensive picture of the tissue composition, Fig. 5(b). The PA image provides a map of the optical properties of the tissue (e.g. blood), while LUS delineates acoustic impedance. The two images are inherently co-registered, because a consistent scanning geometry is employed, and both images are reconstructed with the same acoustic velocity model. The LUS image also aids the interpretation of the PA image. The strong PA signal indicated by the arrow in Fig. 5(a) may be interpreted as a unique optical absorber. However, the composite PA and LUS image in Fig. 5(b) elucidates that the feature is in fact a reflection artifact caused by scattering of the PA wave by the calcium deposit. Nonconfocal LUS (or synthetic aperture) acquisition is also the ideal geometry for removing reflection-artifacts in PA data using techniques such as Marchenko imaging [52] or PAFUSion [53].

We have shown that nonconfocal LUS imaging creates superior images compared to a confocal LUS imaging approach. However, the acquisition and reconstruction times for nonconfocal imaging are increased 100-fold (for 100 sources) compared to confocal imaging. For *in vivo* applications, a confocal scan could be acquired to obtain an initial image and locate target areas. Subsequently, the scan region can be restricted to the concerning section, and a nonconfocal scan can be acquired to obtain a more focused, resolved LUS image. Future studies can optimize the number of sources used to obtain an optimal trade-off between acquisition time and resolution.

LUS imaging provides the same image contrast as ultrasound imaging, but utilizes all-optical hardware. Therefore, LUS is not exempt from the challenges associated with acoustic heterogeneity known to traditional US. Acoustic turbidity of the background medium is not considered in this study, but will degrade the images in *in vivo* imaging scenarios. The adverse affects of acoustic heterogeneity are well-known to traditional US imaging, and include degradation in resolution and increased attenuation of sound in tissue. Here, we have compared nonconfocal LUS, confocal LUS, and CT, and demonstrated the advantages of combining PA and LUS imaging in this complex tissue model. Future studies may assess the axial and lateral resolution achievable with nonconfocal LUS when the sample is covered by a layer of acoustically heterogeneous soft tissue.

The CT scan revealed that the calcification was located outside of the LUS imaging plane (Fig. 4(d)). As a result, the deposit maps to a deeper location in the LUS images than we observe in histology. Instead of a 1D line scan, a 2D scan of the tissue surface could be obtained, and the corresponding 3D images can be reconstructed to accurately image the 3D tissue volume [54]. While the 2D image provides an indication of the location of calcification within the artery wall, a 3D image would account for out-of-plane scattering and therefore accurately locate plaque deposits. While this is feasible in *ex vivo* studies, significant enhancement of the acquisition speed would be required for *in vivo* applications. To reduce the time burden for acquisition, parallelized detection, a source laser with faster repetition rates, or compressed sensing [55] can be implemented.

We have focused on developing imaging techniques for high-resolution, repeatability, and flexibility for non-invasive applications. However, intravascular imaging will be required when frequency-dependent attenuation does not allow imaging with the necessary resolution non-invasively. The advantages of broadband all-optical LUS may still be valuable in this case and is under development [[56], [57]].

Finally, all-optical systems have the potential to be extended to quantitative PA and LUS imaging. Quantitative photoacoustic tomography is an active area of research, with a primary focus on recovering the optical absorption coefficient [58]. Quantitative acoustic amplitudes are required [59], yet non-trivial to obtain in both biomedical PA and (L)US imaging. All-optical systems are most promising to achieve this, because of the quantitative nature of the detectors, as well as the independence of amplitude measurements on user factors. Reverse-time migration is well-suited to quantitative LUS imaging. Future work will also look at joint PA and LUS reconstruction using full-waveform inversion [60].

## 5 Conclusions

We present all-optical laser-ultrasound imaging of the layers of the artery wall and calcification in an excised human carotid artery, and demonstrate the capabilities for combining with photoacoustic imaging. Nonconfocal acquisition improves the resolution and focusing power and reduces artifacts compared to confocal laser-ultrasound imaging. Furthermore, the laser-ultrasound image aids in the interpretation of the photoacoustic image, and helps to identify photoacoustic signals that correspond to reflection artifacts. Upon comparison with state-of-the-art x-ray computed tomography imaging, we observed improved sensitivity to the artery wall and lateral resolution with nonconfocal laser-ultrasound. At the same time, we maintain the advantage of experimental repeatability by using all-optical acquisition that is independent of user variability known to transducer-based ultrasound.

## Conflicts of interest

The authors declare that there are no conflicts of interest.

## Figures and Tables

**Fig. A.6:**
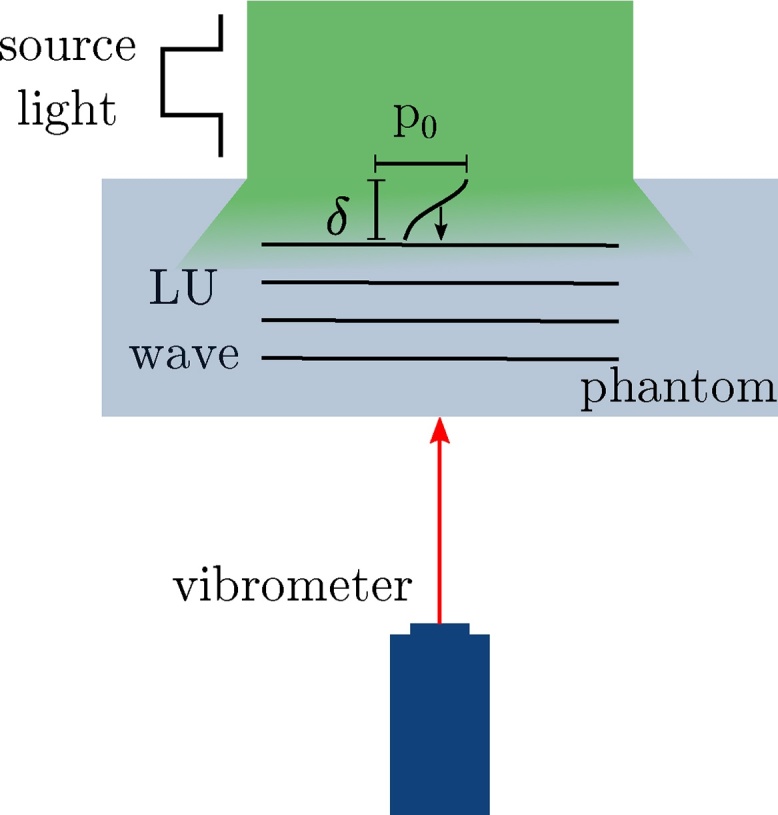
Setup for laser-ultrasound generation experiment. A pulsed laser generates LUS waves at the surface of the tissue, which propagate through the phantom and are recorded on the opposite surface with a laser-Doppler vibrometer.

**Fig. A.7:**
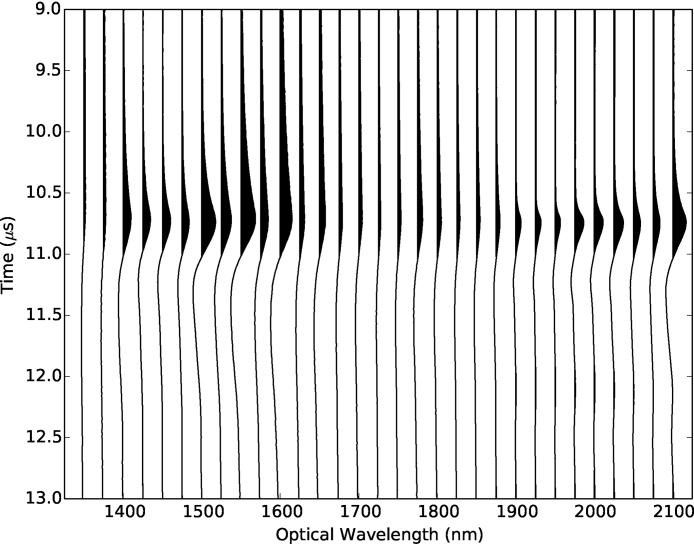
Laser-ultrasound waveforms recorded in the range of 1350–2100 nm, indicating a clear variation in amplitude and frequency with wavelength. The positive peaks are filled to highlight this effect.

**Fig. A.8:**
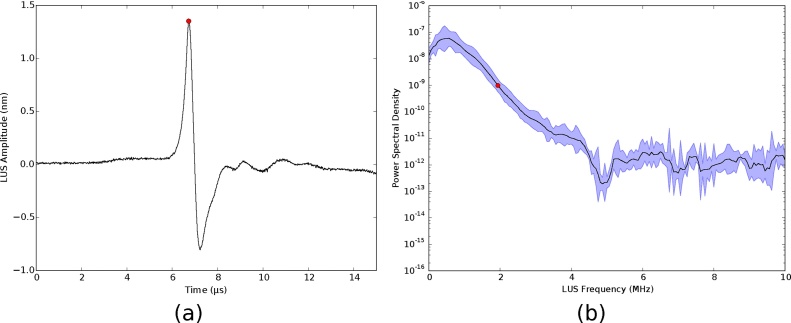
Measurements of laser-ultrasound waveforms at 1450 nm. (a) The raw laser-ultrasound waveform is shown with the maximum amplitude indicated by a red dot. (b) Power spectral density shown with 95% confidence intervals in blue. The mean frequency is indicated by a red dot.

**Fig. A.9:**
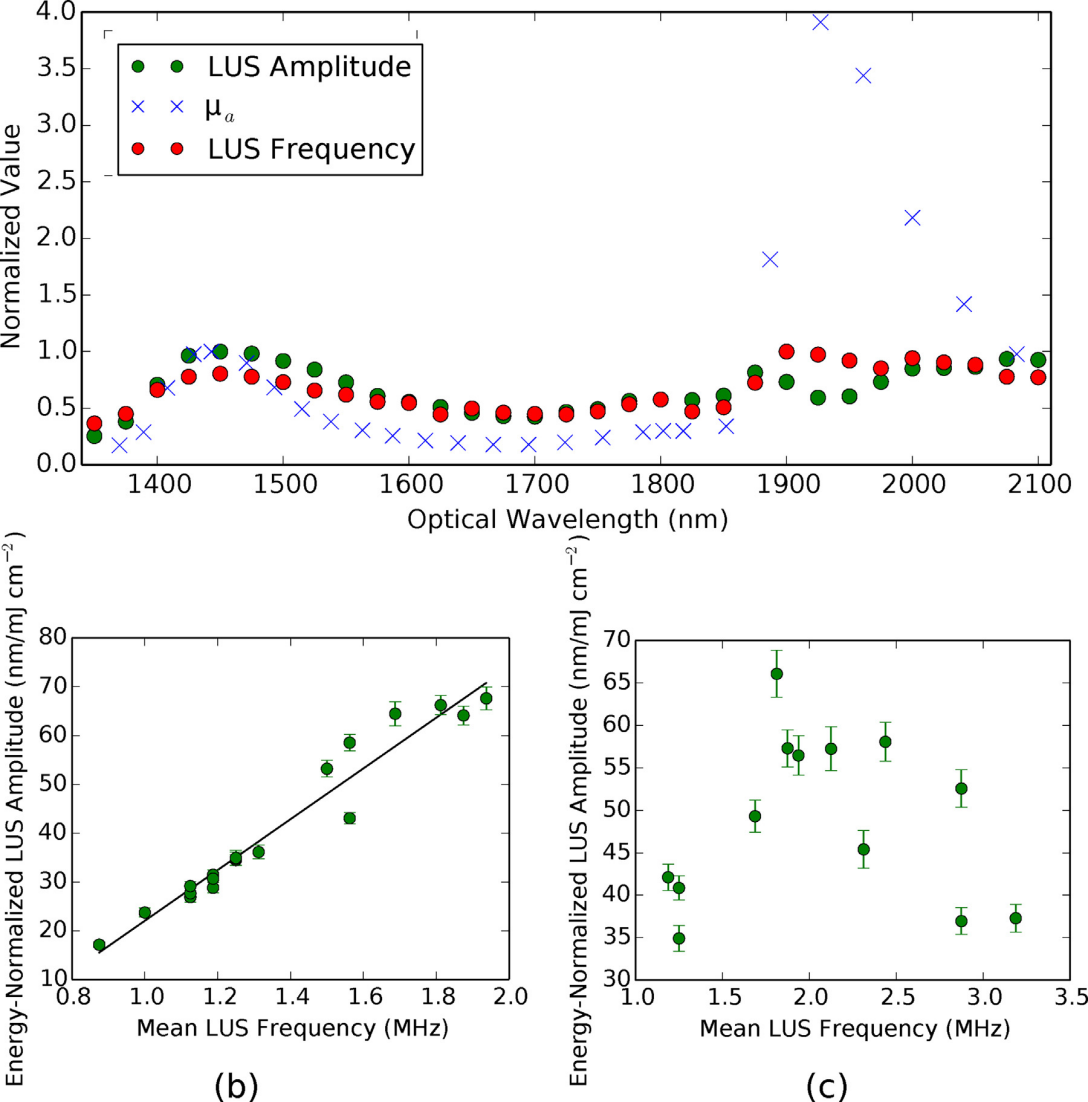
(a) Optical absorption coefficient [45] shown with laser-ultrasound amplitude and mean frequency, where all data are normalized to the value in the corresponding measurement at 1450 nm. (b) Maximum laser-ultrasound amplitude is linearly proportional to the mean laser-ultrasound frequency in the wavelength range of 1350–1800 nm (*R* = 0.97). (c) In the range of 1800–2100 nm, this linearity breaks down due to attenuation of high acoustic frequencies. The error bars are calculated by the percent error in the source laser energy measurement (one standard deviation).

**Fig. A.10:**
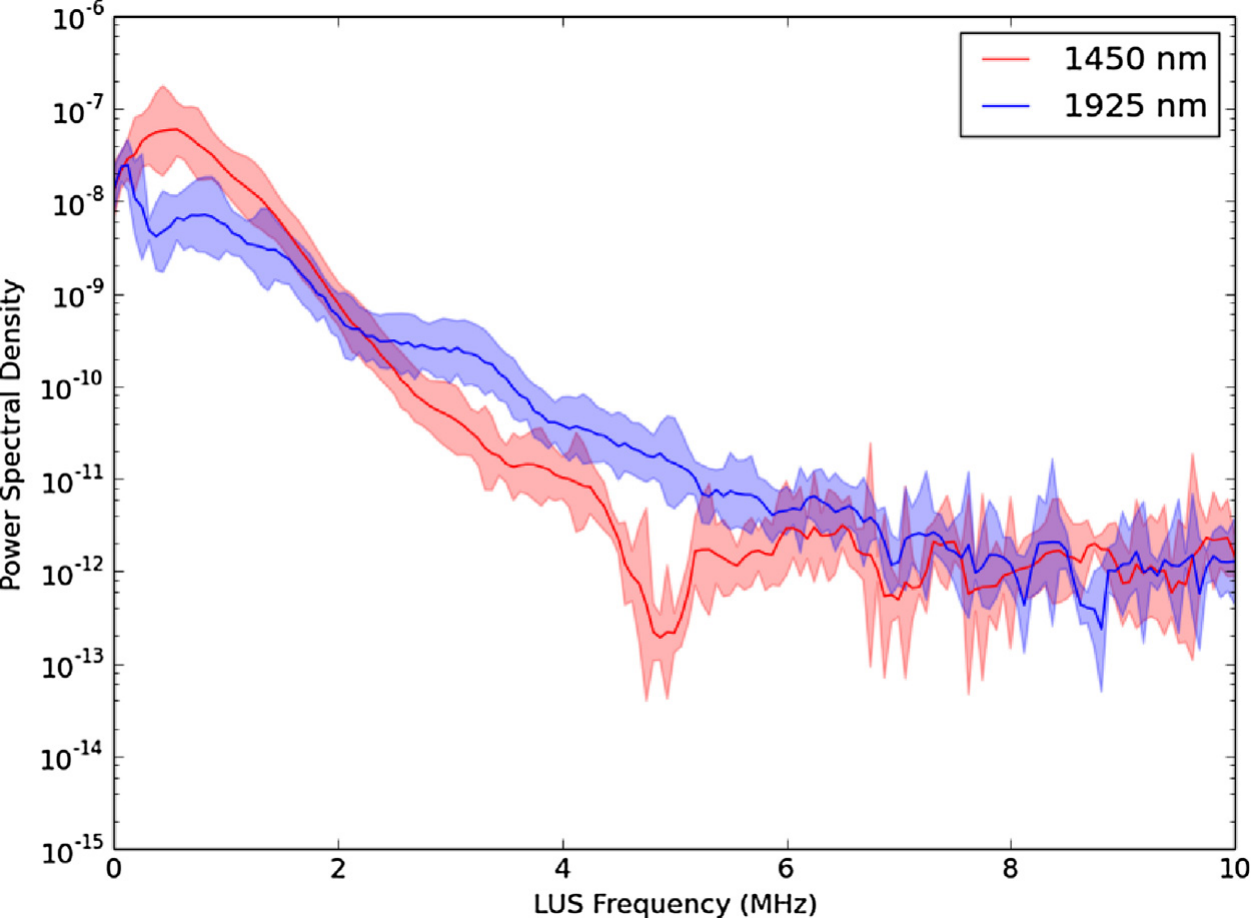
LUS frequency spectrum of wavelengths with the highest absorption coefficient. We observe a trade-off between power and frequency. In the range of 2.25–6 MHz, more power is created by generation with a 1925 nm source than 1450 nm. However, the strongest power is generated in the range of 0–2.25 MHz with a 1450 nm source wavelength.

## Appendix A Optimum wavelength for laser-ultrasound generation in water-rich tissue

The primary constituent of biological cells is water. Therefore, understanding the effects of the source wavelength on the properties of LUS waves generated in water is transferable across a range of tissue types and applications. For deep-tissue imaging, the balance between amplitude (pressure) and frequency become particularly important. Higher amplitude waves will penetrate deeper into tissue, allowing for greater imaging depths; whereas high acoustic frequencies allow high-resolution images.

The initial pressure *p*_*o*_(*r*) generated by the photoacoustic effect is proportional to the optical absorption coefficient *μ*_*a*_(*r*) in the tissue,

(A.1) $$p_{0}(r)=\Gamma \mu_{a}(r)\phi (r;\mu_{a},\mu_{s},g),$$

where Γ is the Grüneisen coefficient of the tissue, *μ*_*s*_ is the optical scattering coefficient, and *g* is the optical anisotropy factor [43]. We can assume that most light is dissipated within a penetration depth (or “skin depth”) *δ* defined by the depth that the optical irradiance has decreased by 1/*e*. This depth is inversely proportional to the effective attenuation coefficient in tissue [43]:

(A.2) $$\mu_{\mathrm{eff}}={\left(3\mu_{a}(\mu_{a}+\mu_{s}^{\prime })\right)}^{1/2}=\frac{1}{\delta },$$

where $\mu_{s}^{\prime }$ is the reduced scattering coefficient.

In the optical window (600–900 nm), water absorption is low, therefore *δ* is large, and PA waves can be generated up to centimeters deep at these wavelengths. Optical wavelengths beyond 1000 nm are strongly absorbed by water, therefore, *δ* is small and LUS generation dominates. The dominant wavelength of a LUS wave is determined by *δ* [61]. In other words, the wavelength of laser-generated waves is proportional to the linear dimension where light is absorbed. Assuming Γ can be considered constant and $\mu_{s}^{\prime }\ll \mu_{a}$ we can deduce that

(A.3) $$p_{0}\propto \mu_{a}\propto \frac{1}{\delta }\propto \nu ,$$

where *ν* = *c*/*λ* is the dominant frequency of the LUS wave. Therefore, for an LUS wave generated by strong optical absorption at the surface of tissue, *p*_0_ and *ν* increase proportionally.

However, acoustic attenuation is frequency-dependent in soft tissue [62]: as *p*_0_ and *ν* increase, so does attenuation. For deep-tissue imaging, a balance must be found between obtaining the highest amplitude LUS wave (within energy safety limits), while maintaining adequate frequency content at depth. It is worth noting that LUS waves are inherently broadband, and the overall bandwidth is beyond 10 MHz. Therefore, the purpose of this experiment is to empirically validate the optical wavelength that will deliver the most overall power at the highest frequencies for deep-tissue imaging.

The tissue phantom used is 1.5 cm thick and composed of 1% agar and distilled water. A tunable OPO laser (Radiant 532 LD, Opotek, Carlsbad, CA, USA) is used to generate LUS waves in the range of 1350–2100 nm in increments of 25 nm at the surface of the phantom. The energy at each wavelength was attenuated to 20 ± 2 mJ/cm^2^ and recorded for each LUS measurement. A laser-Doppler vibrometer (OFV-505, Polytec, Irvine, CA, USA) is focused on retroreflective tape on the opposite surface to record the LUS waves (Fig. A.6). The source beam is unfocused, with a diameter of 5 mm and a Gaussian profile. At each wavelength, the average of 100 LUS waves are recorded with a 100 megasamples per second sampling rate.

The measurements at each wavelength are shown in Fig. A.7. For each waveform, the time series from 0 to 15 μs is analyzed. The multitaper spectral estimate is computed with confidence intervals [63], and the maximum amplitude is extracted as shown in Fig. A.8. We divide the LUS amplitude by the measured source laser fluence to account for the effects of *ϕ* on *p*_0_.

We normalize the LUS amplitude and frequency measurements by the corresponding value at 1450 nm, and plot alongside the normalized optical absorption coefficient of water [45] in Fig. A.9. As expected, both the amplitude and mean frequency of the LUS wave follow a trend matching the absorption coefficient in the range of 1350–1800 nm. Furthermore, the proportionality of the LUS amplitude and frequency are confirmed in this range of wavelengths, corresponding to mean frequencies in the 0–2 MHz range, Fig. A.9(b). However, in the range of 1800–2100 nm, the correlation between LUS amplitude, frequency, and optical absorption coefficient break down (Fig. A.9(c)). Light is absorbed by a greater amount in this wavelength range, which corresponds to higher frequency content and larger amplitudes. According to the known absorption coefficient of water, the LUS amplitude generated is expected to be largest at 1925 nm. However, the measured LUS amplitude recorded by the vibrometer is lower than at 1450 nm. We can see from the power spectral density of the LUS waves that there is a higher mean frequency recorded at 1925 nm compared to 1450 nm, confirming that stronger absorption occurred (Fig. A.10). Therefore, the reduced amplitude at 1925 nm can be attributed to attenuation of the higher LUS frequencies through 1.5 cm of tissue. To maintain a high amplitude for imaging at depths greater than 1.5 cm, a wavelength of 1450 nm is preferred.

For waves propagating less than 15 mm (corresponding to 75 mm imaging depths for reflection-mode LUS), 1925 nm may be advantageous. With sufficient detection sensitivity, the higher frequency LUS waves will allow a higher-resolution image. However, for greater imaging depths, 1450 nm provides greater power (higher amplitude) at the dominant frequency. Furthermore, the proportionality between amplitude and frequency at 1450 nm may provide additional information for quantitative LUS.

We have empirically shown that the optimum wavelength for generating LUS in a water-agar sample for deep-tissue imaging in the range of 1350–2100 nm is 1450 nm. At this wavelength, the mean frequency is a local maximum of 2.25 MHz. At 1925 nm, the mean frequency content is increased to 3 MHz due to a high *μ*_*a*_ and therefore smaller skin-depth. However, this higher-frequency wave is more attenuated, resulting in a lower amplitude wave after propagating through 1.5 cm of the sample than the wave generated at 1450 nm.
